# A Case Report of Mania and Psychosis Five Months after Traumatic Brain Injury Successfully Treated Using Olanzapine

**DOI:** 10.1155/2017/7541307

**Published:** 2017-06-12

**Authors:** Giordano F. Cittolin-Santos, Jesse C. Fredeen, Robert O. Cotes

**Affiliations:** ^1^Faculty of Medicine, Federal University of Rio Grande do Sul (UFRGS), Porto Alegre, RS, Brazil; ^2^Department of Psychiatry and Behavioral Sciences, Emory University School of Medicine, Atlanta, GA, USA

## Abstract

**Background:**

There are few published pharmacologic trials for the treatment of acute mania following traumatic brain injury (TBI). To our knowledge, we present the first case report of an individual being treated and stabilized with olanzapine monotherapy for this condition.

**Case Presentation:**

We describe the case of a 53-year-old African American male admitted to an inpatient psychiatric hospital with one month of behavioral changes including irritability, decreased need for sleep, hyperverbal speech, hypergraphia, and paranoia five months after TBI. Using Diagnostic and Statistical Manual of Mental Disorders, 5th edition (DSM-5) criteria, he was diagnosed with bipolar disorder due to traumatic brain injury, with manic features. He was serially evaluated with clinical rating scales to measure symptom severity. The Young Mania Rating Scale (YMRS) score upon admission was 31, and the Clinician-Rated Dimensions of Psychosis Symptom Severity (CRDPSS) score was initially 9. After eight days of milieu treatment and gradual titration of olanzapine to 15 mg nightly, his symptoms completely abated, with YMRS and CRDPSS scores at zero on the day of discharge.

**Conclusion:**

Olanzapine was effective and well tolerated for the treatment of mania following TBI.

## 1. Introduction

Mania develops in 1.9–9% of individuals after experiencing traumatic brain injury (TBI) [1, 2]. Yet, there is a dearth of literature on pharmacologic treatment options for mania following TBI. The guidelines for the pharmacologic treatment of neurobehavioral sequelae of traumatic brain injury, published in 2006, concluded there was insufficient evidence to support the development of standards or guidelines in the treatment of TBI related mania [3]. Four studies were included in the review, including three case series (each with two patients) and one case report. Treatments successful in treating mania included clonidine [4], thioridazine/amitriptyline [5], electroconvulsive therapy [5], lithium [6], and valproate [6]. In addition to the studies presented in the 2006 guidelines, successful trials have been published with carbamazepine/lithium [7], lithium/thioridazine [8], lithium monotherapy [9–11], valproate/olanzapine [12–14], valproate monotherapy [15], carbamazepine/chlorpromazine [16, 17], haloperidol [16], haloperidol/chlorpromazine [18], haloperidol/clonazepam [19], and quetiapine [20]. Here, we present the first case report of successful treatment with olanzapine monotherapy for mania after a traumatic brain injury.

## 2. Case Presentation

A 53-year-old African American male was brought by Emergency Medical Services (EMS) to the Emergency Room (ER) of an urban, public teaching hospital, due to threatening behavior, irritability, and an inability to care for himself. During the initial psychiatric consultation in the ER, the patient was hyperverbal with pressured speech and a tangential thought process. His mood was elevated, and his affect was labile with sudden and inappropriate bouts of tearfulness. He endorsed decreased need for sleep over the past few days and paranoid and persecutory delusions regarding strange noises around his apartment and his brother stealing money from his father. Per the EMS report, the patient was also emailing and texting neighbors paranoid and threatening messages, which resulted in multiple crisis hotline calls and the patient being brought to the hospital.

His past medical history was significant for a depressive episode treated successfully 25 years ago with sertraline and TBI five months prior to presentation. After the injury, he was followed by an outpatient neurologist for postconcussive syndrome. A brain MRI was ordered three months after TBI, which showed signs of mild white matter small vessel ischemic changes, but no other significant findings. The initial ER workup included a urinalysis, urine drug screen, complete metabolic panel, and thyroid function, all of which were unremarkable. A noncontrast CT scan of the brain was obtained and was unremarkable. He was admitted for inpatient psychiatric hospitalization. Using Diagnostic and Statistical Manual of Mental Disorders, 5th edition (DSM-5) criteria [21], he was diagnosed with bipolar disorder due to traumatic brain injury, with manic features.

The patient was started on olanzapine 2.5 mg by mouth at bedtime upon admission. Manic features remained prominent, as he continued to demonstrate decreased need for sleep (three to five hours per night), pressured speech, irritability, emotional dysregulation, and labile affect. He often arose early in the morning and spent multiple hours writing questions for his treatment team. He refused valproate and lithium despite the team's suggestions. Olanzapine was gradually titrated and reached 15 mg on hospital day (HD) 6. Aside from one instance of refusal on HD 5, he was adherent with olanzapine throughout the hospitalization. Olanzapine was well tolerated. He was evaluated with serial clinical rating scales which included the Young Mania Rating Scale (YMRS) and the Clinician-Rated Dimensions of Psychosis Symptom Severity (CRDPSS). On HD 1, the YMRS score was 31 and CRDSS was 9. On HD 6, the YMRS score decreased to 30 and CRDPSS decreased to 7. On HD 7, he displayed significant clinical improvement. He slept throughout the night and on interview no longer had pressured speech. His thought process was logical, linear, and goal-directed, and the psychotic symptoms had also fully abated. On HD 8, the YMRS and CRDPSS scores were 0. He was subsequently discharged from hospital with appropriate outpatient follow-up.

## 3. Discussion

Mania due to TBI is a challenging diagnosis to make with confidence, and this can make the limited research challenging to interpret. TBI may be an independent risk factor for the development of bipolar disorder [1, 22–24] and the DSM-5 does not report a definitive time course for which the diagnosis of TBI must take place and symptoms must emerge in order for the disorder to be characterized as mania due to TBI [21]. As in our case, the majority of individuals who ultimately develop bipolar disorder report a depressive episode first [25], but the later age of onset of manic symptoms and temporal relationship to TBI lends credence to the TBI's primary role in the development of manic symptoms. In the case presented here, the patient exhibited a combination of manic and psychotic symptoms, but manic symptoms were predominant. The duration of the manic episode (likely around 2 months) was within range of other manic episodes reported after TBI. In study of six patients following head injury who experienced mania, the duration of the episode was 2 months, and the mean estimated duration of elevated mood was 5.7 months [2].

Olanzapine is a second-generation antipsychotic medication effective for the treatment of acute bipolar mania [26] and recommended for acute mania by various guidelines across the world [27–29]. However, few pharmacologic (and no randomized) trials exist for the treatment of mania following TBI. Olanzapine has been used for acute mania following TBI in several reported cases. Grenne et al. [30] described a case of a 13-year-old boy treated with olanzapine 10 mg daily and zonisamide who had improvement of auditory hallucinations but continued mania and delusions. A 60-year-old man was treated with an unspecified dose of olanzapine and 2500 mg of valproate five months after a head injury [12]. A 42-year-old man was treated with olanzapine 15 mg daily and valproate 1000 mg daily for mania emerging three years after a head trauma [13]. A 69-year-old man eighteen months after TBI was treated with 7.5 mg of olanzapine and 250 mg of valproate three times daily [14]. In each of these cases, olanzapine was combined with another medication, and of note, valproate alone has been effective in treating mania secondary to TBI [15], making it challenging to know if the patient improved related to olanzapine or valproate. Furthermore, olanzapine monotherapy has been shown to be effective in treating psychotic symptoms following traumatic brain injury in two case reports [31, 32]. In the case presented here, the patient was not agreeable to other pharmacologic treatments despite being offered lithium and valproate augmentation. In a 2014 review article, Jorge and Arciniegas recommended valproate or quetiapine as first-line therapies for bipolar disorder due to TBI [33]. We conclude that olanzapine could also be considered for this population, as it was effective and well tolerated in this case.
